# Multi-View Human Activity Recognition in Distributed Camera Sensor Networks

**DOI:** 10.3390/s130708750

**Published:** 2013-07-08

**Authors:** Ehsan Adeli Mosabbeb, Kaamran Raahemifar, Mahmood Fathy

**Affiliations:** 1 Computer Engineering Department, Iran University of Science and Technology, Narmak, Tehran 16846-13114, Iran; E-Mail: eadeli@iust.ac.ir; 2 Department of Electrical and Computer Engineering, Ryerson University, 350 Victoria Street, Toronto, ON M5B 2K3, Canada

**Keywords:** human activity recognition, camera sensor networks, consensus, convex optimization, matrix completion, nuclear norm

## Abstract

With the increasing demand on the usage of smart and networked cameras in intelligent and ambient technology environments, development of algorithms for such resource-distributed networks are of great interest. Multi-view action recognition addresses many challenges dealing with view-invariance and occlusion, and due to the huge amount of processing and communicating data in real life applications, it is not easy to adapt these methods for use in smart camera networks. In this paper, we propose a distributed activity classification framework, in which we assume that several camera sensors are observing the scene. Each camera processes its own observations, and while communicating with other cameras, they come to an agreement about the activity class. Our method is based on recovering a low-rank matrix over consensus to perform a distributed matrix completion via convex optimization. Then, it is applied to the problem of human activity classification. We test our approach on IXMAS and MuHAVi datasets to show the performance and the feasibility of the method.

## 1. Introduction

**Figure 1.**
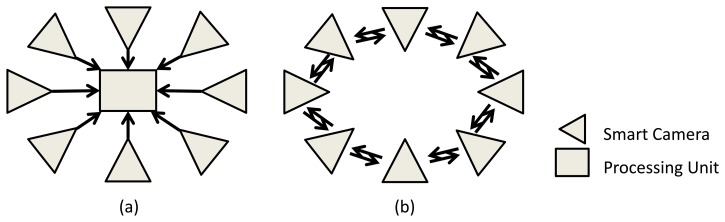
(**a**) Centralized camera network setup; (**b**) distributed camera network setup.

A camera sensor network (CSN) is defined as a set of vision sensors, which can communicate through a network. Each of these smart camera nodes also has its own processing element and memory. With such settings, many applications could be addressed, due to the ease of deployment and their robustness. For instance, creating smart homes, intelligent environments and robot coordination are some great potential applications, which can lead us to a better quality of life. Traditional systems make each camera transmit its own image data or low-level features over the network to a centralized processing unit, which analyzes everything in a centralized fashion (Figure 1(a)). However, this needs a huge amount of processing and communication and requires dealing with a large amount of data. To address this problem, we can develop distributed algorithms, in which each camera deals with its own image (data) and communicates with other cameras in the network. To analyze the whole scene, the cameras collaborate and come to a decision together via fusing their own local analysis (Figure 1(b)) [1,2].

Human action recognition has been proven to have many applications, including vision-based surveillance [3,4], human-computer interaction [5], patient and healthcare monitoring systems [6], smart homes and environments [7] and a lot more [8,9]. This makes it a very important field in computer vision studies. With the development of smart camera technology and networks, the huge amount of processing for such high level applications could be performed in a more robust and scalable way. Several previous works have developed many computer vision applications in such distributed environments [1]. Some also have targeted the activity recognition problem [10–12].

Understanding the events and activities of humans in video sequences is a challenging task, due to several different issues, including: (1) the large variability in the imaging conditions, as well as the way different people perform a particular action; (2) the background clutter and motion; (3) the high dimensionality of such data is another significant challenge for recognition problems; and (4) a huge amount of occlusion in real-world environments. Many previous works have targeted these challenges by introducing different sets of features [13,14] and classifiers and have achieved good results. One of the best methods to overcome many of these challenges is to analyze the activities from multiple views and, therefore, acquire more information about the activity for better understanding. However, this makes it even harder, since there will be more amounts of data to be processed, and on the other hand, the fusion of the information across the views is a hard task. Therefore, camera sensor networks could create a great bed for such applications, where the processing could be distributed among the cameras and the decision about the scene could be made in a distributed manner via communication and fusion of features.

Rank Minimization has recently gained a lot of attention, due to the simple, effective success in solving many problems. As noted by [15], the minimization of the rank function can be achieved using the minimizer obtained by the nuclear norm, which is calculated as the sum of singular values. In the field of computer vision, nuclear norm minimization has been applied to many problems, such as camera calibration [16], structure from motion [17], image segmentation [18] and image categorization [19].

**Figure 2.**
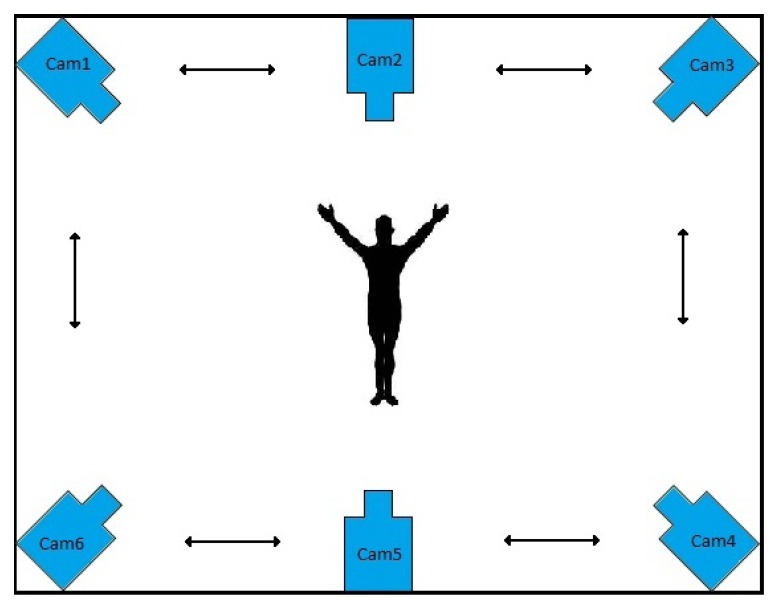
Sample camera network setup for human activity recognition.

In this paper, we develop a method for the recognition of human activities portrayed in multi-view video sequences. Our method is based on matrix completion, which finds the best action label(s) for each test scene. Each view is composed of a single smart camera, which locally processes its video sequence and decides about the activity being performed in the scene via communication. A sample configuration of the smart cameras for activity recognition is depicted in Figure 2. Each scene is represented with a number of fixed length histograms of densely sampled features, which captures both the visual content and the temporal changes in the scene. This makes the method independent from the video content, view point and imaging conditions. In real applications, there is a lot of clutter and noise present in the scene, from the background and/or the imaging conditions, besides the variability in performing the actions by the subjects. Our low-rank matrix recovery framework is capable of taking out the noise and the outliers, efficiently. In this paper, a consensus-based distributed algorithm for matrix completion is presented and is applied for activity recognition in camera sensor networks. The algorithm is based on singular value thresholding to minimize the nuclear norm and enjoys a convex formulation. The minimization problem is solved via the Alternating Direction method of Multipliers (ADM) [20].

In the rest of the paper, the next section reviews the previous work, Section 3 explains our distributed matrix completion technique and the proceeding section explains the proposed activity recognition approach in detail. Section 5 outlines a set of experiments for distributed activity recognition. Finally, Section 6 concludes the paper.

## 2. Related Works

Action and activity recognition methods from single-view video sequences could be categorized into three classes: (1) models that directly utilize bag-of-words (BoWs) representations [21,22]; (2) approaches that decompose an action into smaller parts for capturing the local spatial or temporal structure of the activity and to better model the interaction between parts [23]; (3) approaches that use the global spatio-temporal templates, such as motion history, spatio-temporal shapes, the human model changing in time or other templates [24]. These approaches try to retain the visual shape and structure of the activity. As shown by Laptev *et al.* [13], compared to simple bag-of-words [21], approaches encoding the spatio-temporal layout of a video using a fixed space-time grid enhance the recognition rates.

Several multi-camera and distributed action recognition approaches have also been proposed in the literature [10–12,25–28], which aimed at extending single-view techniques for the multi-view case. Sirvastava *et al.* [10] use spatio-temporal interest points from each single view. This method is specifically designed for a network of low-powered camera sensors. Song *et al.* [11] use a Markov chain with a known transition matrix to model the actions. There are also several papers proposing fusion strategies for multi-view action recognition [29]. Wu *et al.* [25] use the best view as a simple strategy for fusion, whereas [12] uses data from all views for the classification task. In [11], the authors use a probabilistic consensus method for fusing the similarity scores of neighboring cameras.

Matrix completion is a great tool for classification purposes, where the instances are classified through convex optimization for best labels and, simultaneously, finding the error and outliers present in the data. The problem of matrix completion and rank minimization is initially a non-convex optimization problem [30,31], which is simply based on factorizing the matrix into two matrices of a rank of at most *r*. However, recently, rank minimization has gained attention and is achieved by using the minimizer obtained with the nuclear norm [15]. In order to solve this convex rank minimization problem, many approaches are developed, such as Iterative Thresholding [15,32], Fixed Point Continuation [33], the Augmented Lagrangian Multipliers method [32] and the Alternating Direction method [34].

Distributed algorithms for matrix factorization and low rank recovery mostly include using parallel or distributed programming models, such as MapReduce and Hadoop. For instance, [35,36] are designed for MapReduce and [37] for the second version of Hadoop. The drawbacks of these models are that they are limited to the restrictive programming models and mostly suffer from run-time overheads. Furthermore, the cluster management is hard, and optimal configuration of the nodes is not obvious. Other approaches in this area include introducing a separable regularization for the nuclear norm, which makes the process distribution much easier [38,39]. These approaches use the Alternating Direction method or Stochastic Gradient Descent approaches for the optimization process. However, these regularizations or approaches that factorize the main matrix into two lower-rank matrices suggest non-convex objectives.

In this paper, a distributed algorithm is proposed, which uses a convex formulation of matrix completion and is applied to the problem of multi-view activity recognition in a network of smart cameras.

## 3. Distributed Matrix Completion

### 3.1. Network Setup

Let's assume that the network of the processing nodes or the smart cameras is modeled with a connected undirected graph, 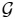 = ( 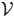, *ε*), with 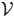 = {1,…, *N_p_*} as the set of camera nodes and *ε* ⊂ 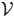 × 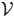 representing the nodes that can communicate with each other. With this definition, each node, *i*, can have some neighbors denoted by 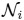 = {*j* ∈ 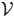: (*i, j*) ∈ *ε*} and the degree, *d_i_* = | 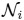
|.

### 3.2. Matrix Completion for Classification

Matrix Completion is the process of recovering a matrix from a sampling of its entries. We want to recover a data matrix, **D**, from a matrix, **D**_0_, in which we only get to observe a number of its entries, which is comparably much smaller than the total number of elements in the matrix. Let Ω denote the set of known entries. With sufficiently large measurements and uniformly distributed entries in the matrix, we can assume that there is only one low-rank matrix with these entries [15]. As denoted by [15,30], if a matrix has rank *r*, it should have exactly *r* nonzero singular values. Thus, the rank function could be simply defined as the number of non-vanishing singular values (*σ_k_*). Therefore, a simple estimate of the rank function can be defined as ${\left\|\text{D}\right\|}_{*}=\sum_{k=1}^{d}\sigma_{k}(\text{D})$, which is called the nuclear or trace norm. Recently, this formulation has been used for classification tasks. The task is to learn the connection between the space of features, *X*, and the space of labels, *Y*, from *N_tr_* training instances. Let *m* be the number of different classes, *n* the dimensionality of the feature space, *N* the number of total instances and *N_tr_* and *N_tst_* the number of training and testing instances, respectively.

**Figure 3.**
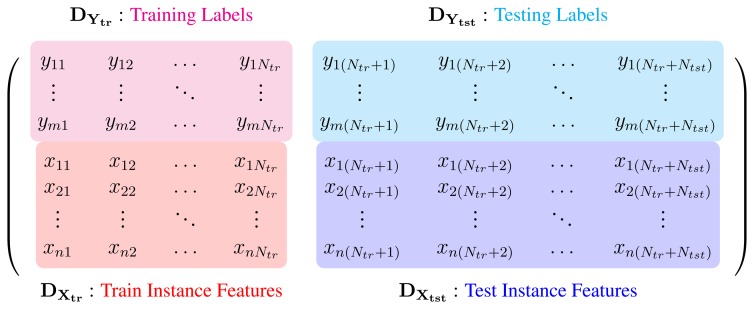
Data matrix, **D**_0_, which contains training and testing instances, each as a single column.

As noted by Goldberg *et al.* [33] the problem of classifying *N_tst_* test entries can be cast as a matrix completion task. To this end, we can concatenate all labels and features into a single matrix (as illustrated in Figure 3). If a linear classification model holds, this matrix should be rank-deficient. In this formulation, the classification process would be defined as filling the unknown entries in *Y_tst_*, such that the nuclear norm of **D**_0_ is minimized. This could be done via a convex minimization process [33,34,40]. In practice, we have errors and incomplete data in the training features and labels. Therefore, we define the set of known entries in **D**_0_ as Ω*_X_* and Ω*_Y_* and zero out unknown entries:

(1) $$\text{D}=\left[\begin{array}{c} {\text{D}}_{\text{Y}}\\ {\text{D}}_{\text{X}}\\ {\text{D}}_{1} \end{array}\right]=\left[\underset{1^{\text{T}}}{\begin{array}{cc} {\text{Y}}_{\text{tr}} & {\text{Y}}_{\text{tst}}\\ {\text{X}}_{\text{tr}} & {\text{X}}_{\text{tst}} \end{array}}\right]+\left[\underset{0^{\text{T}}}{\begin{array}{cc} {\text{E}}_{{\text{Y}}_{\text{tr}}} & 0\\ {\text{E}}_{{\text{X}}_{\text{tr}}} & {\text{E}}_{{\text{X}}_{\text{tst}}} \end{array}}\right]$$

where **Y_tr_** ∈ ℝ^*m*×*N*_*tr*_^ and **Y_tst_** ∈ ℝ^*m*×*N*_*tst*_^ are the training and testing labels and **X_tr_** ∈ ℝ^*n*×*N*_*tr*_^ and **X_tst_** ∈ ℝ^*n*×*N*_*tst*_^ are the training and testing feature vectors, respectively. Therefore, the classification process would be posed as finding the best **Y_tst_** and the error matrix, **E**, such that the rank of **D** = **D**_0_ + **E** is minimized [33]. This would be equivalent to [40]:

(2) $$\begin{array}{ll} \min_{\text{D}} & \gamma {\left\|\text{D}\right\|}_{*}+\frac{1}{\left|\Omega_{X}\right|}\sum_{ij\in \Omega_{X}}c_{x}({\text{E}}_{{\text{X}}_{\text{ij}}})+\frac{\lambda_{1}}{\left|\Omega_{Y}\right|}\sum_{ij\in \Omega_{Y}}c_{y}({\text{E}}_{{\text{Y}}_{\text{ij}}})\\ \text{subject to} & \begin{array}{l} \text{D}={\text{D}}_{0}+\text{E},\\ {\text{D}}_{1}=1^{\text{T}} \end{array} \end{array}$$

where *c_y_*(.) is a log loss function and *c_x_*(.) is a least squares error. These two terms are to avoid trivial solutions and to penalize large distortions of **D**. The parameters, *γ* and λ_1_, are positive trade-off weights [33,40]. This minimization problem can be solved using a Fixed Point Continuation (FPC) method [33] or an Alternating Direction method (ADM) [34].

### 3.3. Distributed Nuclear Norm Minimization for Matrix Completion

As shown by [33,41], as long as the error matrix, **E**, is sufficiently sparse, we can exactly recover the low-rank matrix, **D**, from **D**_0_ = **D** + **E** by solving the convex optimization problem, Equation (2). Let us, for simplicity, replace the second and the third terms in the objective function in Equation (2) with *f*(**E_X_**) and *g*(**E_Y_**), respectively. By introducing a Lagrangian multiplier, the Lagrangian function would be:

(3) $$L(\text{D},\text{E},ℒ)=\gamma {\left\|\text{D}\right\|}_{*}+f({\text{E}}_{\text{X}})+g({\text{E}}_{\text{Y}})++\langle ℒ,{\text{D}}_{0}-\text{D}-\text{E}\rangle +\frac{\mu }{2}{\left\|{\text{D}}_{0}-\text{D}-\text{E}\right\|}_{F}^{2}$$

Using the iterative thresholding or the singular value thresholding (**SVT**) algorithm [41,42] and the Alternating Direction method, Problem (2) could be solved by updating each variable, while keeping the others fixed. **D** and **E** are calculated by minimizing 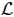
(**D**, **E**, ℒ), and then, the amount of violation, **D**_0_ — **D** — **E**, is used to update ℒ. A shrinkage operator as a proximal operator for the nuclear norm could be defined as:

(4) $$S_{\epsilon }[x]=\{\begin{array}{ll} x-\epsilon & \text{if}\;x>\epsilon \\ x+\epsilon & \text{if}\;x>-\epsilon \\ 0 & \text{otherwise} \end{array}$$

With the singular value decomposition of a matrix, **USV**^T^, we can apply an Alternating Direction method (ADM) for recovering the low-rank matrix, **D**, via an iterative optimization procedure, as proposed by [32,41]. For this purpose, we need to iterate to optimize the above Lagrangian function for the **E** and **D** matrices. The error matrices, **E_X_** and **E_Y_**, would have a closed form solution, by solving the following two subproblems in each *k^th^* iteration:

(5) $$\begin{array}{l} {\text{E}}_{{\text{X}}_{\text{k}+1}}=\underset{{\text{E}}_{\text{X}}}{\mathrm{argmin}}\frac{1}{\mu_{k}}f({\text{E}}_{{\text{X}}_{\text{k}}})+\frac{1}{2}{\|{\text{E}}_{{\text{X}}_{\text{k}}}-({\text{D}}_{0\text{X}}-{\text{D}}_{{\text{X}}_{\text{k}+1}}+\frac{{ℒ}_{k}}{\mu_{k}})\|}_{F}^{2}\\ {\text{E}}_{{\text{Y}}_{\text{k}+1}}=\underset{{\text{E}}_{\text{Y}}}{\mathrm{argmin}}\frac{1}{\mu_{k}}g({\text{E}}_{{\text{Y}}_{\text{k}}})+\frac{1}{2}{\left\|{\text{E}}_{{\text{Y}}_{\text{k}}}-({\text{D}}_{0\text{Y}}-{\text{D}}_{{\text{Y}}_{\text{k}+1}}+\frac{{ℒ}_{k}}{\mu_{k}})\right\|}_{F}^{2} \end{array}$$

where *μk* is the step parameter and is increased in each iteration. On the other hand, the nuclear norm of the matrix, **D**, is minimized using the SVT algorithm [42], where the proximal operator, 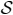
_ϵ_[.], is applied on the singular values of the matrix, ${\text{D}}_{0}-{\text{E}}_{\text{k}}+\mu_{k}^{-1}{ℒ}_{k}$, to construct the matrix, **D**, in each *k^th^* iteration as:

(6) $$\begin{array}{r} \left(\text{U},\text{S},\text{V})=\text{svd}({\text{D}}_{0}-{\text{E}}_{\text{k}}+\mu_{k}^{-1}{ℒ}_{k}\right)\\ {\text{D}}_{k+1}={\text{U}S}_{\mu_{k}^{-1}}\left[\text{S}\right]{\text{V}}^{\text{T}}. \end{array}$$

The constraint, **D**_1_ = **1**^T^, is enforced by keeping the last row of **E_k_** equal to **0**^T^. Furthermore, for all unknown entries, (*i*, *j*) ∈ Ω*_Y_*, the choice of **E_k_**(*i*, *j*) = 0 holds [32].

In order to parallelize this algorithm, we need to distribute the entries present in **D**_0_ between the processing nodes. Therefore, we will have separate **E** matrices for each node, and accordingly, we will require the use of the corresponding Lagrangian multipliers. Suppose that we split the data matrix, **D** ∈ ℝ^(*n*+*m*)×(*N*_*tr*_+*N*_*tst*_)^, into *N_p_* parts, **D_i_** ∈ ℝ^*n*_*i*_×(*N*_*tr*_+*N*_*tst*_)^. Therefore, we can assume that the original data matrix is formed as:

(7) $$\text{D}={\left[{{\text{D}}_{1}}^{\text{T}},{{\text{D}}_{2}}^{\text{T}},\cdots ,{{\text{D}}_{{\text{N}}_{\text{P}}}}^{\text{T}}\right]}^{\text{T}}\in {\mathbb{R}}^{\left(n+m)\times (N_{tr}+N_{\text{tst}}\right)}.$$

Therefore, the Lagrangian multiplier, ℒ, and the error matrix, **E**, would also be split in the same manner. Now, we will have an equivalent problem, as in Equation (2), for each single processing node, *i.* The Lagrangian function, from each node's point of view, would be:

(8) $$\gamma {\left\|\text{D}\right\|}_{*}+f({\text{E}}_{{\text{X}}_{\text{i}}})+g({\text{E}}_{{\text{Y}}_{\text{i}}})+\langle {ℒ}_{i},{\text{D}}_{\text{i}}-{\text{D}}_{0_{\text{i}}}-{\text{E}}_{\text{i}}\rangle +\frac{\mu }{2}{\left\|{\text{D}}_{\text{i}}-{\text{D}}_{0_{i}}-{\text{E}}_{\text{i}}\right\|}_{F}^{2}$$

where ℒ*_i_*s are the Lagrange multipliers. The only shared problem between the nodes is the minimization of the nuclear norm of the whole data matrix, where we need to calculate the SVDof the $\text{J}={\text{D}}_{0}-{\text{E}}_{\text{k}}+\mu_{k}^{-1}{\text{Y}}_{\text{k}}$ matrix, collaboratively. First, suppose we want to compute $\frac{1}{N_{p}}{\text{J}}^{\text{T}}\text{J}$:

(9) $$\text{C}=\frac{1}{N_{p}}{\text{J}}^{\text{T}}\text{J}=\frac{1}{N_{p}}\sum_{i=1}^{N_{p}}{{\text{J}}_{\text{i}}}^{\text{T}}{\text{J}}_{\text{i}}=\frac{1}{N_{p}}\sum_{i=1}^{N_{p}}{\text{C}}_{\text{i}}.$$

${\text{C}}_{\text{i}}={{\text{J}}_{\text{i}}}^{\text{T}}{\text{J}}_{\text{i}}$ could be denoted as the local correlation matrix. As could be seen, this problem would be distributed on the nodes. This is very easy to compute through consensus, since it is a simple averaging of data present in each node. Initially, each node has a local state, **c_i_**(0) = **C**_i_; in each iteration, nodes receive the internal state of their neighbors and update:

(10) $${\text{c}}_{\text{i}}(t+1)={\text{c}}_{\text{i}}(t)+W(t)\sum_{j\in N_{i}}\left({\text{c}}_{\text{j}}(t)-{\text{c}}_{\text{i}}(t)\right)$$

where 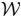
(*t*) is initially set to (*max_i_*{*d_i_*})^−*1*^ and decreased through time. It is shown [43] that each state converges to the average of the initial values in each node (limt_t→∞_ **c_i_** = **C**), no matter how the network configuration is and if there is partial noise in the communications. The consensus would be achieved when |c_i_(*t* + 1) − c_i_(*t*)| ≤ *ϵ*, with *ϵ* as a very small constant threshold.

Note that **C** is a (*n* + *m*) × (*n* + *m*) matrix, independent from *N_p_*. Therefore, if the number of processing nodes and the number of data splits grow, **C** still could be correctly recovered. In order to compute the SVD of the matrix, **J**, we need to calculate matrices, **U** ∈ ℝ^(*n*+*m*)×*r*^, **V** ∈ ℝ^*r*×(*N*_*tr*_+*N*_*tst*_)^ and Σ ∈ ℝ^*r*×*r*^, with *r* as the rank of the matrix: **J** = **UΣV**^T^. To do this, we can compute the SVD of **C**, which would be equal to $\text{V}\left(\frac{1}{N_{p}}\sum^{2}\right){\text{V}}^{\text{T}}$. Therefore, after distributed averaging, each node can recover **V**, and if they know *N_p_*, they also can recover Σ. These two matrices will be common for all the nodes and easy to calculate, and they can compute their own share of the matrix, **U** as: **U_i_** = **J_i_VΣ**^−1^.

As a result, the **SVD** operation could be calculated in a distributed manner, and each node can recover the complete matrix, **Σ**, and then it can apply the shrinkage operator and iterate to optimize the rank of the data matrix. In order to minimize the rank of the matrix, **D**, in each *k^th^* iteration, the following set of instructions should be executed on each node, *i*, until a consensus is achieved:

(11) $$\begin{array}{l} {\text{J}}_{{\text{i}}_{\text{k}}}={\text{D}}_{{\text{i}}_{\text{0}}}-{\text{E}}_{{\text{i}}_{\text{k}}}+\mu_{k}^{-1}{\text{Y}}_{{\text{i}}_{\text{k}}}\\ {\text{C}}_{\text{i}}={{\text{J}}_{{\text{i}}_{\text{k}}}}^{\text{T}}{\text{J}}_{{\text{i}}_{\text{k}}}\\ \text{calculate C via consensus using Equation}(10),\\ \left(\text{V},\frac{1}{N_{p}}\sum^{2},{\text{V}}^{\text{T}})=\text{svd}(\text{C}\right)\\ {\text{U}}_{\text{i}}={\text{J}}_{{\text{i}}_{\text{k}}}\text{V}\sum^{-1}\\ {\text{D}}_{{\text{i}}_{\text{k}+\text{1}}}={\text{U}}_{\text{i}}S_{\tau }\left[\sum \right]{\text{V}}^{\text{T}} \end{array}$$

In summary, this algorithm consists of two stages: first, calculating ***C*** via consensus over the network and, then, performing the iterative thresholding algorithm for minimizing the nuclear norm. The first stage is performed by iterating on Equation (10), while receiving the local *C_i_* variables from the neighboring nodes, in each iteration. This is continued until the *C_i_* variables converge. We can benefit from a joint treatment and create an inexact version of the algorithm, where the iterative operations for calculating the ***C****_i_*s is not performed completely to reach the convergence. Only one iteration gives us a fast good estimate of the ***C*** matrix and would satisfy the convergence properties of the whole algorithm. When a good estimate could be achieved for the optimization subproblem, ADM would still converge, probably with more numbers of iterations [32]. The distributed matrix completion algorithm on each processing node, *i*, is outlined in Algorithm 1.


**Algorithm 1** Distributed matrix completion algorithm for recognition, on the *i^th^* processing node.
**Input:** Initial portion of the data matrix for the *i^th^* node, **D**_i_ = **D**_0_**_i_**, and parameter, λ.**Output:**
*i^th^* portion of the completed matrix, **D_i_** ℒ*_i0_*, = 0, *μ_k_* > 0, *ρ* = 1.1, **E**_i_0__ = **0** **while** not converged **do**  1. Fix all other variables and update 
${\text{D}}_{{\text{i}}_{\text{k}+\text{1}}}=\underset{{\text{D}}_{i}}{\mathrm{argmin}}\frac{1}{\mu_{k}}{\left\|{\text{D}}_{{\text{i}}_{\text{k}}}\right\|}_{*}+\frac{1}{2}{\left\|{\text{D}}_{{\text{i}}_{\text{k}}}-({\text{D}}_{0_{\text{i}}}+{\text{E}}_{{\text{i}}_{\text{k}}}-\frac{{ℒ}_{{\text{i}}_{\text{k}}}}{\mu_{k}})\right\|}_{F}^{2}$  by:   
${\text{J}}_{{\text{i}}_{\text{k}}}={\text{D}}_{{\text{i}}_{\text{0}}}-{\text{E}}_{{\text{i}}_{\text{k}}}+\mu_{k}^{-1}{ℒ}_{{\text{i}}_{\text{k}}}$   c_i_(*k*) = **J_i_k__**^T^
**J_i_k__**   Send c_i_(*k*) to all the neighbors, 

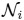
,   Receive all c_j_(*k*)s from the neighbors, 

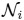
,   c_i_(*k*) = c_i_(*k*) + 

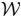
(*k*) Σ_*j*∈

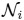
_(c_j_(*k*) − c_i_(*k*))   (**V**, 
$\frac{1}{N_{p}}\sum^{2}$, **V**^T^) = *svd*(c_i_(*k*))   **U_i_** = **J_i_k__ VΣ**^−1^   **D_i_k+1__**= **U_i_**

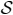
_τ_[**Σ**] **V**^T^  2. Fix all other variables and update  
${\text{E}}_{{\text{X}}_{{\text{i}}_{\text{k}+\text{1}}}}=\underset{{\text{E}}_{{\text{X}}_{\text{i}}}}{\mathrm{argmin}}\frac{1}{\mu_{k}}f({\text{E}}_{{\text{X}}_{{\text{i}}_{\text{k}}}})+\frac{1}{2}{\left\|{\text{E}}_{{\text{X}}_{{\text{i}}_{\text{k}}}}-({\text{D}}_{{\text{0X}}_{\text{i}}}+{\text{D}}_{{\text{X}}_{{\text{i}}_{\text{k}+\text{1}}}}-\frac{{ℒ}_{{\text{i}}_{\text{k}}}}{\mu_{k}})\right\|}_{F}^{2}$  3. Fix all other variables and update  
${\text{E}}_{{\text{Y}}_{{\text{i}}_{\text{k}+\text{1}}}}=\underset{{\text{E}}_{{\text{Y}}_{\text{i}}}}{\mathrm{argmin}}\frac{1}{\mu_{k}}g({\text{E}}_{{\text{Y}}_{{\text{i}}_{\text{k}}}})+\frac{1}{2}{\left\|{\text{E}}_{{\text{Y}}_{{\text{i}}_{\text{k}}}}-({\text{D}}_{{\text{0Y}}_{\text{i}}}+{\text{D}}_{{\text{Y}}_{{\text{i}}_{\text{k}+\text{1}}}}-\frac{{ℒ}_{{\text{i}}_{\text{k}}}}{\mu_{k}})\right\|}_{F}^{2}$  4. Set the 
${\text{E}}_{{\text{i}}_{\text{k}}}={\left[\begin{array}{lll} {{\text{E}}_{{\text{Y}}_{{\text{i}}_{\text{k}}}}}^{\text{T}} & {{\text{E}}_{{\text{X}}_{{\text{i}}_{\text{k}}}}}^{\text{T}} & 0^{\text{T}} \end{array}\right]}^{\text{T}}$,  5. Update the multiplier, ℒ*_i_*: ℒ_*i*_*k*+1__ = ℒ_*i*_*k*__ + μ*k*(**D**_i_*k*+1__ − **D_i_0__** − **E_i_k+1__**)  7. Update parameter, *μ_k+1_*, as: *μ_k+1_=* min(*ρμ_k_*, 10^10^) and *k* = *k* + 1.  8. Check the convergence condition: (**D_i_k__** − **D_0_i__** −**E_i_k__** → 0) **end while**


## 4. Distributed Activity Recognition

Our task is to recognize activities present in the scene, which are captured with a networked set of cameras, as also illustrated in Figure 2. The distributed environment, as described in Section 3.1, is composed of a number of cameras with processing power and communication skills. Each scene is represented with a fix-length feature vector from each camera's view point. The recognition task would be to classify these feature vectors into one of the predefined activity classes. This is performed in a distributed manner via consensus, as will be described in this section.

### 4.1. Scene Representation

To represent each video from each view, we use histograms of densely sampled features, which extract features from space-time video blocks and sample from five dimensions, (*x*, *y*, *t*, *σ*, *τ*). *σ* and *τ* are the spatial and temporal scales, respectively. We use a histogram of gradient (HoG) and a histogram of optical flow (HoF) [13]. These histograms are computed on a regular grid at three different scales. For each descriptor (HoG, HoF), an independent dictionary is used. This is done by using K-means and quantizing all descriptors to the closest ℓ2 distance dictionary element. The concatenation of both histograms forms the scene descriptor from a camera's view point. These histogram features have been extensively used for object and activity recognition in a single view [8,23] and also extended for multi-view [10]. With these feature vectors, there is no need to perform any background subtraction, tracking or silhouette extraction, which makes the algorithm faster and independent from contextual noise. As a result, each scene, *i*, is composed of a histogram feature vector, ${\text{h}}_{\text{i}}^{\text{j}}$, from the *j^th^* view. Therefore, scene **S_i_** is described by $\left\{{\text{h}}_{\text{i}}^{1},{\text{h}}_{\text{i}}^{2},\cdots ,{\text{h}}_{\text{i}}^{{\text{N}}_{\text{c}}}\right\}$. These sets of features are almost independent from variations in the activity orientation. However, in order to further make sure that the orientation of the activities with regard to the cameras does not strengthen noise and outliers, we employ a cycling approach, as proposed by [10]. This is explained in more detail in the next subsection.

### 4.2. Training and Testing Scenarios

**Figure 4.**
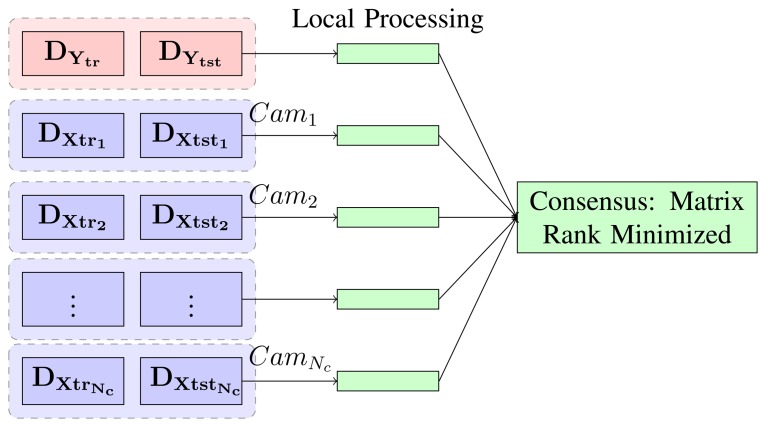
A model for the data split between the processing camera nodes (distributing segments of each activity between the nodes).

We can assume that both train and test action sequences are captured by *N_c_* cameras. With the above representation, each scene is described with a histogram of quantized features from each view. Therefore, each camera has its own part of the scene description. We can model the distribution of the data matrix, **D**_0_ for our case, as shown in Figure 4. The data matrix (as in Equation (1)) is split between the processing nodes, row-wise. Each node will hold one part of the data segment (both train and test). The label's sub-matrix (upper row in Figure 4) is also assigned to a single node.

We construct the matrix, **D**_0_, by assigning each column to training or testing samples. During the process of capturing the sequences of each action, the subject could be facing any of the cameras performing the action. For training, the samples are formed, such that all the sequences have the same orientation formation. Therefore, in order to enhance the recognition results, for each test sequence, we need to determine the orientation for which the action can best perform the recognition. The correspondence could be determined using a circular shift. For instance, consider an action scene, ${\text{s}}_{\text{i}}=\{{\text{h}}_{\text{i}}^{1},{\text{h}}_{\text{i}}^{2},{\text{h}}_{\text{i}}^{3},{\text{h}}_{\text{i}}^{4}\}$, in case of four camera views. The circularly shifted versions are: $\left\{{\text{h}}_{\text{i}}^{1},{\text{h}}_{\text{i}}^{2},{\text{h}}_{\text{i}}^{3},{\text{h}}_{\text{i}}^{4}\right\}$, $\left\{{\text{h}}_{\text{i}}^{4},{\text{h}}_{\text{i}}^{1},{\text{h}}_{\text{i}}^{2},{\text{h}}_{\text{i}}^{3}\right\}$, $\left\{{\text{h}}_{\text{i}}^{3},{\text{h}}_{\text{i}}^{4},{\text{h}}_{\text{i}}^{1},{\text{h}}_{\text{i}}^{2}\right\}$ and $\left\{{\text{h}}_{\text{i}}^{2},{\text{h}}_{\text{i}}^{3},{\text{h}}_{\text{i}}^{4},{\text{h}}_{\text{i}}^{1}\right\}$, which cover all possible conditions, where the action may face any of the cameras.

When performing a matrix completion, for determining the labels, all four combinations are considered, and the one with the least amount of absolute error in the corresponding row of the error matrix, **E_X_**, is chosen, and the action class would be determined by its corresponding column in **D_Y_**.

## 5. Experiments

In this section, we setup several experiments on some well-known multi-view activity datasets and compare the recognition results with some state-of-the-art distributed and centralized methods. We choose previous methods, which have reported results with the same experimental setup for comparisons. We also compare the execution times of our distributed matrix completion algorithm with those of the original centralized version of the algorithm, solving Equation (2) using ADM, on the same datasets. The recognition accuracies are calculated as the average of per-class recognition rates, for each experiment. Recognition results for each single view are also computed by running a matrix completion scheme on the features from that specific view.

### 5.1. Human Action Datasets

**Figure 5.**
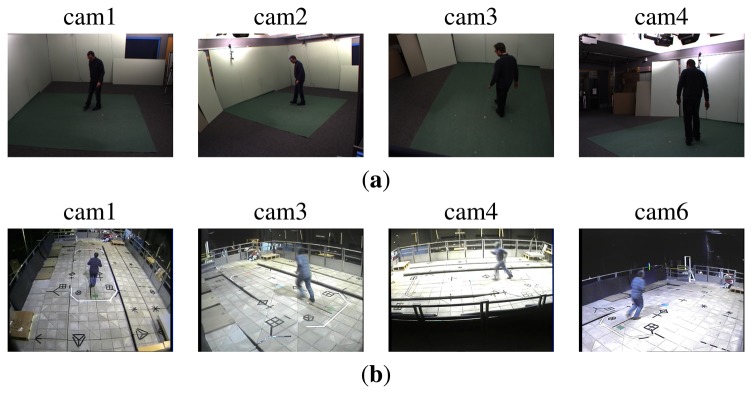
Sample frames from the action datasets. (**a**) IXMAS; (**b**) MuHAVi.

In order to validate our approach, we carried out experiments using the IXMAS [44] and MuHAVi [45] datasets. Figure 5 shows some sample frames from these datasets.

The IXMAS dataset has 13 action classes (check watch, cross arms, scratch head, sit down, get up, turn around, walk, wave, punch, kick, point, pick up, throw over head and throw from bottom up) performed by 12 subjects, each 3 times. The scene is captured by 5 cameras, and the calibration/synchronization parameters are provided. In order to be consistent with a setup similar to those in the previous work [10,44], we discard images from camera 5, which is the top view and does not have much informative information for our purpose. This dataset is a challenging one, due to the fact that subjects freely choose their position and orientation. Therefore, each camera has captured different viewing angles, which makes the recognition task harder.

The MuHAVi dataset contains 17 action classes (walk turn back, run stop, punch, kick, shotgun collapse, pull heavy object, pickup throw object, walk fall, look in car, crawl on knees, wave arms, draw graffiti, jump over fence, drunk walk, climb ladder, smash object and jump over gap) performed by 7 actors, recorded in 25 fps with challenging lighting conditions. In our experiments, we choose four (two side and two corner) cameras for evaluations. A manually annotated subset (MuHAVi-MAS) is also available, which provides silhouettes for two of these views (front-side and corner) for two actors, labeled 14 (called MuHAVi-14). We run our experiments on the whole dataset, since we did not require the manually annotated silhouettes, but we compare our method with some state-of-the-art methods on MuHAVi-14.

### 5.2. Experimental Setup

To setup this experiment, we have simulated the network environment, where each camera process is implemented in a single process on a processing core of a Corei7-3610QM CPU, and the communication is done via IPC. The network of the cameras is considered to have a fully connected topology.

For extracting the spatio-temporal interest points and to form the histogram feature vectors, we set *σ* = 2 and *τ* = 3. For the feature extraction phase, the size of the space-time patches are considered to be 18 × 18 pixels and 10 frames. The samplings are also done with 50% overlap, as also introduced by [46]. For evaluating the experiments, the leave-one-out cross-validation strategy is employed, where videos of one subject are used for testing, and videos of the remaining subjects are considered as the training instances.

### 5.3. Results

#### IXMAS

**Figure 6.**
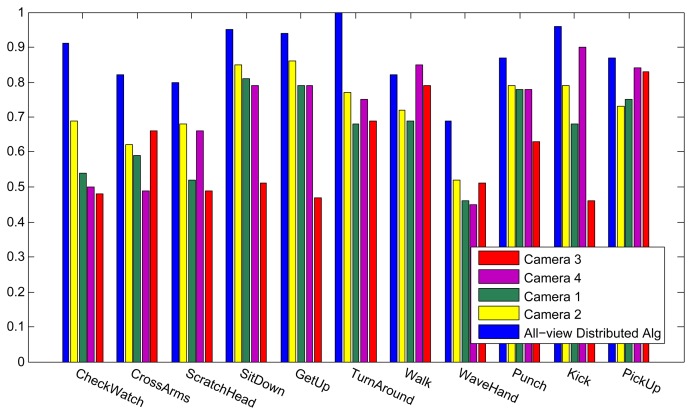
Recognition results for each of the single views and all four views, on the IXMAS dataset with training and testing on 11 actions and 10 subjects.

**Figure 7.**
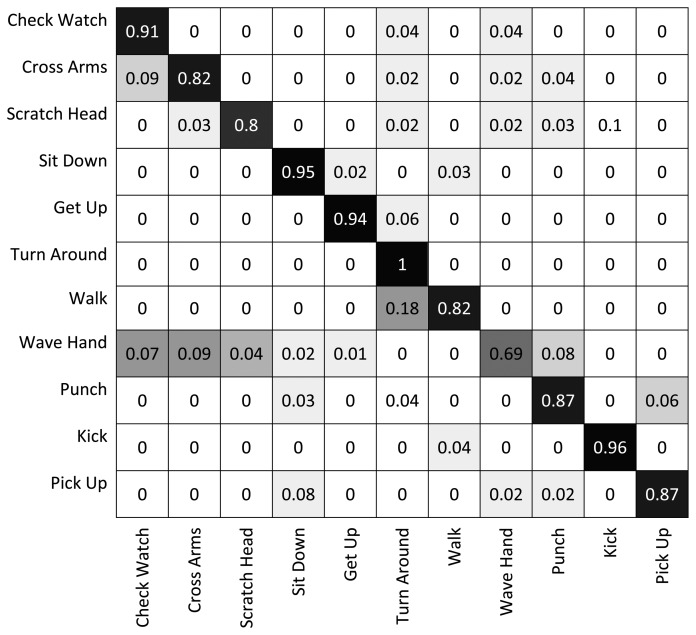
The confusion matrix of the recognition output on the IXMAS dataset.

**Figure 8.**
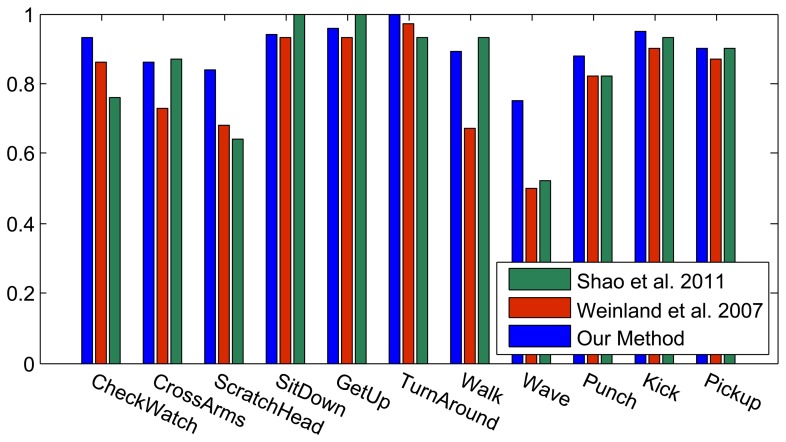
Class-level recognition results of the IXMAS dataset with 11 actions, in comparison with Shao *et al.* [50] and Weinland *et al.* [44].

**Figure 9.**
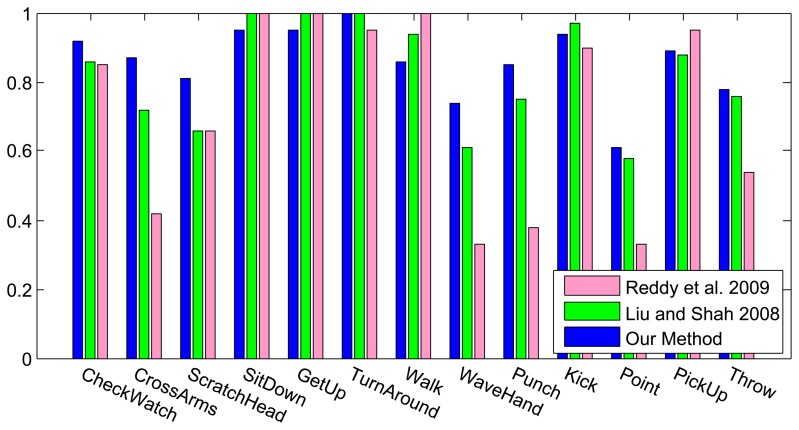
Class-level recognition results of the IXMAS dataset with 13 actions, in comparison with Reddy *et al.* [48] and Liu and Shah [47].

Figure 6 shows the results of the classification on each individual camera for the IXMAS dataset, compared with the distributed algorithm that uses the data from all the views. This figure shows how the distributed algorithm can outperform each of the single views, and that is because it can describe each action in a more descriptive way from different views. Figure 7 outlines the confusion matrix of the distributed activity recognition, and Table 1 shows the overall recognition rate in comparison with some state-of-the-art methods. As is obvious, the WaveHand action is the most deceptive one and could be mistaken with other actions. Different experiments from different previous work use 11 or all 13 actions from the dataset. We run our method and report results on both. Figures 8 and 9 also show the class-level recognition accuracies in comparisons with some state-of-the-art methods. As could be seen in these figures, our method has better recognition rates, even for those actions that are not well-recognized by other competitors.

#### MuHAVi

**Figure 10.**
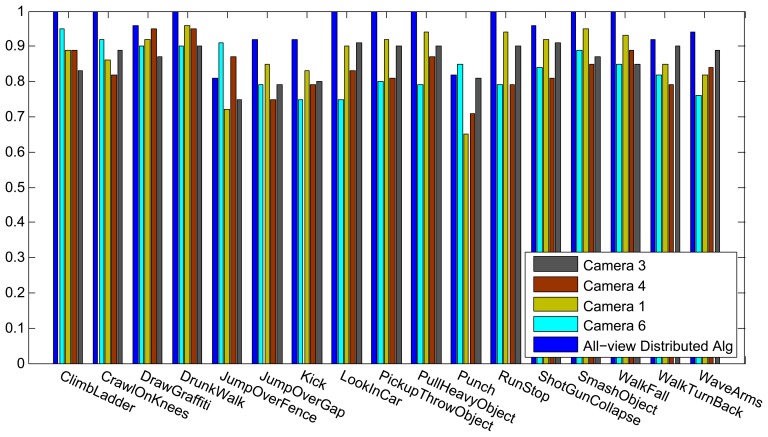
Recognition results for each of the single views and all four views, on the MuHAVi dataset.

**Figure 11.**
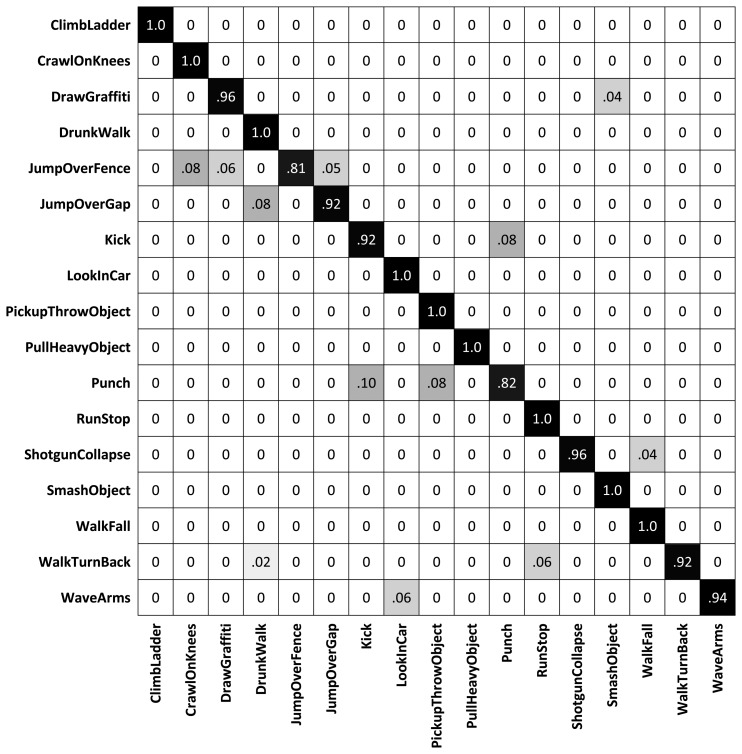
The confusion matrix of the recognition output on the MuHAVi dataset.

**Figure 12.**
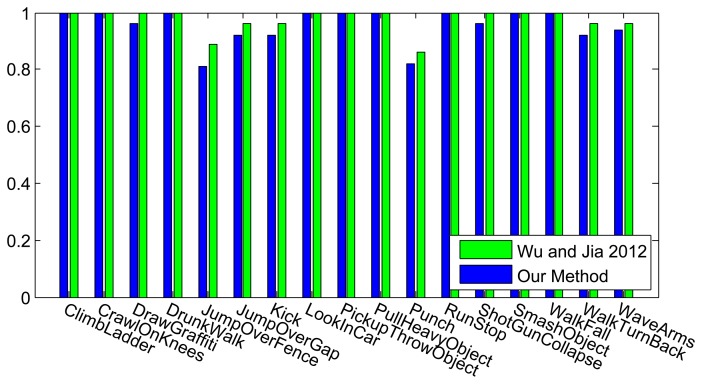
Class-level recognition results of the MuHAVi dataset, in comparison with Wu and Jia [49].

The classification results for every individual camera using our method, in comparisons with our distributed algorithm, are shown in Figure 10, and as expected, the distributed algorithm achieves better recognition results. In this figure, the results for each camera indicate a training and testing scenario on that single view, while the all-view method trains and tests our distributed algorithm. The confusion matrix is also plotted in Figure 11 and the overall recognition rate in comparison with some state-of-the-art methods is shown in Table 2. A class-level comparisons with another state-of-the-art method is provided in Figure 12. This dataset is not as challenging as the IXMAS dataset, since the subjects are not performing the actions freely. The subjects perform the actions with predefined orientations. That is why our method and most of the previous methods get better recognition results on this dataset, compared to the IXMAS dataset.

Many actions are very hard to recognize if they are viewed from a specific view point. However, our distributed algorithm achieves better recognition rates, compared to each single view of the same dataset. As could be seen, our method outperforms several distributed or centralized methods, both as an overall recognition system or in the class-level. Only Wu and Jia [49] achieve better results on these datasets. They use a non-linear classification method with a specific kernel designed for view-invariant classification, while our method enjoys a linear classification scheme, which is capable of being adapted for any large-scale or distributed classification problem.

**Figure 13.**
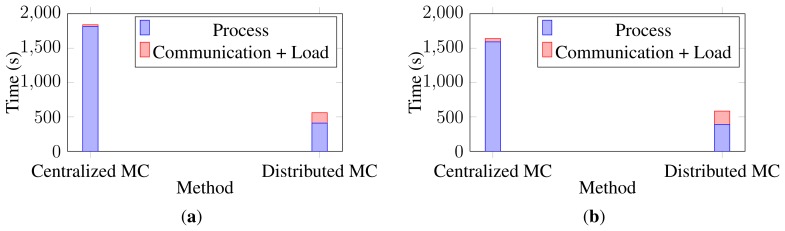
Execution times for the distributed and centralized matrix completion on human activity recognition datasets. (**a**) IXMAS Dataset; (**b**) MuHAVi Dataset.

In order to evaluate the boost in the run time, the execution times of the runs on the two versions of the algorithm are calculated. Figure 13 shows the execution time of each set of data together with the communication and load overheads. As is obvious, the distributed algorithm gets the same recognition results in a shorter time, as expected. The centralized matrix completion algorithm is run on the same machine in which the distributed algorithm was simulated, but on a single core. Note that these reported execution times do not include the circular shifting between the cameras.

## 6. Conclusion and Discussions

In this paper, we have described a distributed action recognition algorithm, based on low-rank matrix completion. We have proposed a simple distributed algorithm to minimize the nuclear norm of a matrix, and then, we have adapted an inexact augmenting Lagrangian multiplier method to solve the matrix completion problem. We have tested the algorithm on IXMAS and MuHAVi datasets and achieved good results. With the experiments outlined in this paper, we show that our matrix completion framework could be well adapted for the classification of a scene in a distributed camera network. Therefore, it is a proof-of-concept study for using such algorithms in distributed computer vision algorithms.

As mentioned before, we have developed a distributed classification framework for human action recognition, which can also be used for distributed classification tasks. Matrix completion is a great tool for dealing with noisy data. As could be seen in the formulations, the error and outliers are identified during the minimization task. Activity recognition data, due to its many variations across subjects and imaging/illumination conditions, is a set of data with many potential outliers, and that is why our method could achieve acceptable results, compared to the other state-of-the-art method.

As a direction for future work, we need to perform the training and testing procedures incrementally, where huge amounts of data could be summarized into smaller matrices and used for testing purposes.

## Figures and Tables

**Table 1.** Overall accuracy results on the IXMAS dataset, using all four cameras. # Sub. and # Act. in the table are the number of subjects and the number of actions taken into account for evaluation in the method, respectively.

**Approach**	**# Act.**	**# Sub.**	**Method**	**Accuracy**
Srivastava *et al.* [10]	10	11	Distributed	81.4%
Weinland *et al.* [44]	10	11	Centralized	81.3%
Our Method	10	11	Distributed	87.5%
Liu and Shah [47]	13	12	Centralized	82.8%
Reddy *et al.* [48]	13	12	Centralized	66.5%
Wu and Jia [49]	12	12	View-invariant	91.67%
Our Method	13	12	Distributed	85.9%

**Table 2.** Overall accuracy results on the MuHAVi dataset. The data column shows the subset of the data used for evaluation for each of the methods.

**Approach**	**Data**	**Method**	**Accuracy**
Singh *et al.* [45]	MuHAVi-14	Centralized	82.4%
Chaaraoui *et al.* [51]	MuHAVi-14	Centralized	91.2%
Wu and Jia [49]	All of the dataset	View-invariant	97.48%
Our method	All of the dataset	Distributed	95.59%
